# Editorial: The Impact of Dietary Changes on Non-communicable Diseases in Latin America

**DOI:** 10.3389/fnut.2022.890873

**Published:** 2022-04-13

**Authors:** Pramil N. Singh

**Affiliations:** Center for Health Research, School of Public Health, Loma Linda University, Loma Linda, CA, United States

**Keywords:** nutrition, obesity, diabetes, trial, diet, overweight

In a 2019 analysis of 479,809 adults from 13 nations in Latin America, Miranda and colleagues (1) reported sharp increases in obesity prevalence during 1998–2017, and that over time, the obesity burden is moving into the lower socioeconomic stratum. Prevention of an obesity burden shift to the lower socioeconomic stratum is identified in the report as a high-impact regional preventive goal.

Popkin and Reardon (2) have primarily attributed the increasing obesity burden in Latin America to a nutrition transition from traditional cultural whole plant foods (i.e., legumes) with minimal processing or refinement of carbohydrates to a diet that is high in (1) animal products, (2) processed/ultra-processed foods composed of refined carbohydrates, high sodium, and saturated fats, and (3) sugar-sweetened beverages.

When conceptualizing a culture-specific plant-based dietary intervention to reverse the nutrition transition in Latin America, we note that the region has a rich cultural tradition of growing, eating, and preparing regional whole plant foods with minimal processing (3–5). For example, the “Three Sisters” diet tradition emerged in Mexico as a companion planting method to optimize the yield of corn, beans, and squash (6). These practices shaped a rural Meso-American diet pattern consisting of simply prepared meals of beans, corn, and squash with minimal processing, and low in refined grains and sugars (4, 5). The Tarahumara Indians of Mexico follow a “Three Sisters” diet pattern, and in a landmark 1991 crossover trial published in New England Journal of Medicine (7), this pattern produced a lower risk of cardio-metabolic disease that was significantly and rapidly reversed by then acculturating the Tarahumara to a typical US “affluent diet.”

In Bolivia, a similar tradition of plant food patterns (maize, beans, and quinoa) from rural indigenous traditions is evident, but in urban and peri-urban areas, indigenous diet is transitioning to more processed foods and animal products (8). WHO STEPS data from Cochabamba, Bolivia (*n* = 10,704) (9) indicate: (1) a high prevalence of abdominal adiposity (54.1%), overweight (35.8%), and obesity (20%), and (2) that the obesity burden may be attributable to a change in lifestyle pattern to physical inactivity and a nutrition transition whereby 76.7% of the population reported low consumption of fruits and vegetables. This growing obesity burden has contributed to obesity-related cancers (Gallbladder, Breast, Colorectum, Liver, Stomach), accounting for more than 35% of all incident cancers in Bolivia (10).

WHO STEPS data from Bolivia (11) also indicate a lower rate of obesity in persons of indigenous ancestry—a trend potentially indicating indigenous lifestyle patterns of higher physical activity and intake of minimally processed plant foods. It is notable that among indigenous communities in remote rural regions of Bolivia are forager-horticulturist groups such as the Tsimane tribe who have long been enrolled in NIH funded studies that have documented how their minimally processed plant-based/plant-forward diets and high levels of physical activity are associated with the lowest coronary artery disease risk scores ever recorded in a human population (12). The Tsimane tribe also exhibits very low rates of other NCD risk factors (obesity, type 2 diabetes, hypertension, hypercholesteremia) (13). The plant-based/plant-forward diet pattern of the Tsimane consists of a high fiber diet of starchy crops (75% of energy from plantain, rice, cassava (manioc), maize) that is supplemented by lean game, freshwater fish, and fruits (14). Interestingly, a panel study of the Tsimane conducted over 10 years did reveal that marginal exposure to market-purchased food products (oil, lard, domesticated meats) was associated with gradual increases in BMI (15). Overall, the cultural traditions of plant-based/plant-forward diets in rural Bolivia provide a rich data source for designing culturally tailored, plant-based/plant-forward diets to reverse the nutrition transition occurring in the nation and region. To date, progress in the academic sector to develop and design such diets has been slow.

In this landmark supplement of Frontiers of Nutrition, the authors from several Latin American nations provide findings that seed a plant-based research agenda for Latin America. Cairo et al. provide findings clearly showing how obesity and overweight have reached the rural areas of Brazil. The emergence of processed and ultra-processed foods in diet patterns across the lifespan in Latin America is shown in pre-schoolers in Chile by Araya et al. and reviewed for the entire region by Matos et al.. Despite these strong trends, there remains a paucity of research infrastructure in Latin America for culturally tailored dietary intervention trials to reverse the nutrition transition away from cultural diets based on minimally processed whole plant foods and fewer animal products. The supplement continues the build of this emergent research infrastructure for dietary intervention. Sanchez Urbano et al. provide evidence of the feasibility and acceptability of dietary intervention advice in the Latin American context. Loureiro et al. provide insights from diet patterns in Brazilian adults, and Contreras-Guillén et al. is innovating dietary recall methods for Argentina. Figueroa et al. tackle the question of whether a plant-based Mediterranean diet can be adapted for the Latin American region. Taken together, the supplement articles herein are a stride forward in the path to reverse the nutrition transition that is creating a sizable non-communicable disease burden in Latin America.

## Author Contributions

The author confirms being the sole contributor of this work and has approved it for publication.

## Conflict of Interest

The author declares that the research was conducted in the absence of any commercial or financial relationships that could be construed as a potential conflict of interest.

## Publisher's Note

All claims expressed in this article are solely those of the authors and do not necessarily represent those of their affiliated organizations, or those of the publisher, the editors and the reviewers. Any product that may be evaluated in this article, or claim that may be made by its manufacturer, is not guaranteed or endorsed by the publisher.
